# Sociodemographic and environmental factors associated with dengue, Zika, and chikungunya among adolescents from two Brazilian capitals

**DOI:** 10.1371/journal.pntd.0011197

**Published:** 2023-03-16

**Authors:** Ana Paula Razal Dalvi, Gerusa Gibson, Alberto Novaes Ramos, Katia V. Bloch, Geziel dos Santos de Sousa, Thiago Luiz Nogueira da Silva, José Ueleres Braga, Marcia C. Castro, Guilherme Loureiro Werneck

**Affiliations:** 1 Sergio Arouca National School of Public Health, Oswaldo Cruz Foundation, (Ensp/Fiocruz), Rio de Janeiro, Brazil; 2 Public Health Institute–IESC, Federal University of Rio de Janeiro–UFRJ, Rio de Janeiro, Brazil; 3 Postgraduate Program in Public Health, School of Medicine, Federal University of Ceará, Fortaleza, Brazil, and Department of Community Health, Faculty of Medicine, Federal University of Ceará, Fortaleza, Brazil; 4 Municipal Health Department of Fortaleza, Ceará, Brazil; 5 Epidemiology Analyst at GSK, Rio de Janeiro, Brazil; 6 Department of Epidemiology, Social Medicine Institute, State University of Rio de Janeiro, Rio de Janeiro, Brazil; 7 Department of Global Health and Population, Harvard TH Chan School of Public Health, Boston, Massachusetts, United States of America; US Department of Homeland Security, UNITED STATES

## Abstract

Among the emerging and reemerging arboviral diseases, Zika, dengue and chikungunya deserve special attention due to their wide geographical distribution and clinical severity. The three arboviruses are transmitted by the same vector and can present similar clinical syndromes, bringing challenges to their identification and register. Demographic characteristics and individual and contextual social factors have been associated with the three arboviral diseases. However, little is known about such associations among adolescents, whose relationships with the social environment are different from those of adult populations, implying potentially different places, types, and degrees of exposure to the vector, particularly in the school context. This study aims to identify sociodemographic and environmental risk factors for the occurrence of Zika, dengue, and chikungunya in a cohort of adolescents from the Study of Cardiovascular Risks in Adolescents—ERICA—in the cities of Rio de Janeiro/RJ and Fortaleza/CE, from January 2015 to March 2019. Cases were defined as adolescents with laboratory or clinical-epidemiological diagnosis of Zika, dengue, or chikungunya, notified and registered in the Information System for Notifiable Diseases (SINAN). The cases were identified by linkage between the databases of the ERICA cohort and of SINAN. Multilevel Cox regression was employed to estimate hazard ratios (HR) as measures of association and respective 95% confidence intervals (95%CI). In comparison with adolescents living in lower socioeconomic conditions, the risk of becoming ill due to any of the three studied arboviral diseases was lower among those living in better socioeconomic conditions (HR = 0.43; 95%CI: 0.19–0.99; p = 0.047) and in the adolescents who attended school in the afternoon period (HR = 0.17; 95%CI: 0.06–0.47; p<0.001). When compared to areas whose Building Infestation Index (BII) for *Aedes aegypti* was considered satisfactory, a BII in the school region classified as “alert” and “risk” was associated with a higher risk of arboviral diseases (HR = 1.62, 95%CI: 0.98–2.70; p = 0.062; HR = 3.72, 95%CI: 1.27–10.9; p = 0.017, respectively). These findings indicate that living in less favored socioeconomic conditions, attending school in the morning, and having a high BII for *Ae*. *aegypti* in school’s region can contribute to an increased risk of infection by Zika, dengue, or chikungunya in adolescents. The identification of residential or school areas based on those variables can contribute to the implementation of control measures in population groups and priority locations.

## Author summary

Zika, dengue, and chikungunya are arbovirus with a wide geographic distribution that can cause serious diseases. The three pathogens are transmitted by the same vector and can present similar symptoms, hindering their identification and register. Despite the great efforts to control arboviruses diseases, Brazil still registers frequently epidemics. Scientific evidence of the association of the environment, vectors, humans, and their behaviors in the arbovirus transmission is vast, but little in know about those association in adolescents. This study focused on adolescents, a part of the population whose relationships with the social environment are different from those of adult and child populations, implying different types, and degrees of exposure to the vector, particularly in the school context. For this reason, this study aims to identify sociodemographic and environmental factors associated with Zika, dengue, and chikungunya disease in adolescents in Rio de Janeiro and Fortaleza municipalities in Brazil from 2015 to 2019. The results obtained in this study highlight factors that may be influencing the transmission of Zika, dengue and Chikungunya, enabling effective measures to be implemented in the school context to control these arboviruses.

## Introduction

Emerging and reemerging communicable diseases are a growing concern for researchers and health managers around the world. An especially troubling example are arboviral diseases like dengue, Zika, and chikungunya, mainly transmitted by the *Ae*. *aegypti* mosquito, a vector widely distributed over tropical and subtropical regions [1]. Brazil has dealt with recurrent epidemics of dengue (DENV) since the 1980s. With the introduction of Zika (ZIKV) and chikungunya (CHIKV) in 2014–2015, the country had to reorganize its health services to face a triple epidemic. ZIKV has become an important event in this context due to its association with cases of microcephaly [2, 3] and Guillain-Barré syndrome [4]. From the introduction of CHIKV in Brazil in 2014 up to May 2019, 589,076 cases were reported, with a greater concentration of cases and deaths in the state of Ceará. In 2018 and 2019, cases were mainly concentrated in the state of Rio de Janeiro, considered the first place to present an important transmission outside the Northeast region. In the same period, 4.5 million presumptive cases of DENV were registered in Brazil, 55,615 of which were severe cases. From 2016 to 2019, when ZIKV was included in the list of notifiable diseases, 239,634 probable cases of ZIKVwere reported in Brazil, with more than 3,000 confirmed cases of Congenital Zika Syndrome [5].

Many factors have been associated with the occurrence of these three arboviral diseases [6]. The most important are sociodemographic factors [7–11] and characteristics of the household and its surroundings [12]. Studies have shown that people in a precarious socioeconomic situation are more affected by DENV[9] and CHIKV [8], but other studies have shown the opposite for ZIKV [13] and DENV [8]. People in low socioeconomic conditions usually live in places with precarious basic sanitation, which can lead to a greater proliferation of mosquitoes and, consequently, to a higher exposure and incidence of these diseases.

Some studies could not demonstrate the association between vector abundance and disease transmission [14, 15], but multiple local factors can interfere in this process, like vegetation cover [16, 17]. Urban environments present a higher concentration of vectors, which are abundantly found inside the households [18]. As a probable consequence, most studies are based on place of residence as a proxy for place of infection. However, studies have found that vectors prefer to feed in the daytime [19, 20], when many people are at the workplace or, in the case of children and adolescents, at school. The identification of *Ae*. *aegypti* that were positive for DENVand ZIKV inside schools strengthens this conception, characterizing as one of the potential places of transmission [21, 22].

Increased knowledge about the dynamics of ZIKV, DENV and CHIKV transmission still coexists with uncertainties about individual and contextual risk factors that can influence the risk of becoming ill. Particularly, little is known about such associations among adolescents, whose relationships with the social environment are different from those of adult populations, implying potentially different places, types, and degrees of exposure to the vector. Filling this gap can enhance knowledge about differences in the risk profile of arboviral diseases in populations belonging to distinct age groups; furthermore, it can contribute to the implementation of control measures at the population level targeted at the peri-domestic and school contexts.

This study aimed to identify sociodemographic risk factors for the occurrence of ZIKV, DENV, and CHIKV in a cohort of adolescents from the Study of Cardiovascular Risks in Adolescents—ERICA—in the cities of Rio de Janeiro/state of Rio de Janeiro (RJ) and Fortaleza/state of Ceará (CE), from January 2015 to March 2019.

## Methods

### Ethical aspects

The ERICA study is conducted according to the principles of the Declaration of Helsinki. The baseline survey, the longitudinal and the present study were approved by the Research Ethics Committee of the Institute of Studies in Collective Health of the Federal University of Rio de Janeiro (report numbers: 3629403, 3437503, and 3645507). Permission to conduct the study was obtained in all State and local Departments of Education and in all schools.

As required by the Research Ethics Committee, written informed consent was obtained from each student and also from their parents for those students who were invited to take blood collection. For those not having their blood collected, a written informed consent was obtained only from the student. However, when the local Departments of Education and schools required informed parental consent even for students who were not taking blood, such parental written consent was also obtained. During the data collection, care was taken to guarantee the student’s privacy and confidentiality, such as when using folding screens for anthropometric measurements.

### Study design and identification of cases of arboviral diseases

This is a cohort study conducted with adolescents from the Study of Cardiovascular Risks in Adolescents—ERICA. ERICA is a nationwide survey conducted in 2013 and 2014 with approximately 85 thousand adolescents aged 12 to 17 years enrolled in public and private schools in Brazilian cities [23].

In our study, cases of ZIKV, DENV, and CHIKV in adolescents were identified through the linkage of the ERICA database with the database of cases of these arboviral diseases from the Information System for Notifiable Diseases (SINAN), in the cities of Rio de Janeiro/RJ and Fortaleza/CE. Cases of arboviral diseases with laboratory or clinical-epidemiological diagnosis of ZIKV, DENV, or CHIKV, and the onset of symptoms from January 2015 to March 2019 were selected. ZIKV, DENV, and CHIKV notification data for the study period were formally requested from the Health Departments of the cities of Rio de Janeiro and Fortaleza.

The probabilistic linkage of data from the city of Rio de Janeiro was performed through two packages of the R software, *RecordLinkage* [24] and *FastLink* [25], to increase sensitivity to detect cases. The variables employed in the linkage were name, date of birth, sex, and residential address of the adolescent, and name of the mother. The data linkage for Fortaleza was performed manually at the Federal University of Ceará (UFC) in partnership with the Health Surveillance Department—Fortaleza Municipal Health Secretariat/CE (Epidemiological Surveillance Coordination Office—CEVEPI).

### Characteristics of adolescents and schools

Information on skin color, age, and sex was extracted from the ERICA baseline survey (2013–2014). Skin color was classified as “white” or “non-white”. The adolescents’ age varied from 12 to 17 years. Data on characteristics of the household, number of rooms in the household, number of people sleeping in the same room, presence of maids working in the household, presence of bathroom, and asset ownership (television, washing machine, DVD player, refrigerator, freezer, computer, Internet access, cars, and motorcycles) were used to construct a socioeconomic indicator by Principal Component Analysis (PCA). The school variables analyzed in the study were type of institution (public or private school) and the period in which the student attended school (morning or afternoon).

The contextual variables at the level of census tracts were obtained from the 2010 Census (Brazilian Institute of Geography and Statistics—IBGE) and linked to the schools attended by the adolescents, considering the census tract in which it was localized [26]. The contextual variables (or variables of the census tract in which the school is located) included the proportions of households with water supplied by the distribution network, sewage collected by the sewerage network, garbage collection, sidewalk, storm drain/manhole, and vegetation cover in the surrounding area. These variables were dichotomized into “High” (≥ 90%) or “Low” (< 90%). The variables ‘proportion of households with open-air sewage’ and ‘garbage accumulated in the surrounding streets’ were categorized as “High” (> 10%) or “Low” (≤ 10%).

The *Aedes aegypti* Infestation Rapid Survey (LIR*Aa*, for its acronym in Portuguese), obtained from the Municipal Health Surveillance Department of Fortaleza/CE and from the Municipal Health Department of Rio de Janeiro/RJ, provided the Building Infestation Index (BII) according to the location of each school in the analyzed strata. BII is the ratio between the number of positive buildings for larvae and pupae of *Ae*. *aegypti* and the number of surveyed buildings (BRASIL, 2009), expressed as a percentage. It is measured in three or four different periods distributed over a year. Rio de Janeiro performed four measures for each year (2014–2018), and in Fortaleza three assessments occurred during 2014–2017 and four only in 2018. For the years in which there were four measures in the municipality, the BII value was redefined to 3 periods as following: a) the first period corresponded to the first BII measurement of the year; b) the second period corresponded to the average of the two measurements performed in the middle of the year; c) the third period corresponded to the last BII measurement of the year. The areas where the schools were located were labelled as “satisfactory” (BII<1), “alert” (1≤BII≤3.9), or “risk” (BII>3.9), according to the classification established by the Brazilian Ministry of Health [27].

The incidence of arboviral disease was calculated with their respective 95% confidence interval (95%CI) for each characteristic. Adolescents per period were used to estimate the incidence by BII, since the BII levels varied through time.

### Data analysis

#### Principal Component Analysis (PCA)

To capture a summary statistic of the adolescents’ socioeconomic condition, PCA was implemented based on information extracted from the ERICA questionnaire answered in 2013–2014.

All variables were categorized according to the reported number of each asset in the adolescents’ households, 0 (zero), 1 (one), 2 (two), 3 (three), and 4 (four or more), except for computers and internet access, which were dichotomized into present (1) or absent (0).

The application of PCA aimed to synthesize the correlations between the analyzed variables and find the linear combination of the original variables where maximum variance in a particular orthogonal dimension is extracted [28]. The *Psych* package of the R software was used separately for each city, employing a varimax rotation. Eigenvalues and variances were obtained for each component. The first extracted component explained 28.6% of the variance in the city of Fortaleza and 24.3% of the variance in the city of Rio de Janeiro (S1 Fig). The weights of the variables were calculated with the values of the coordinates of each variable, extracting only the first factor (S1 Table) and multiplying them by each value of each variable for each observation. These values were added and then divided into quintiles [29], corresponding to five levels of socioeconomic conditions: “Very High”, “High”, “Medium”, “Low”, and “Very Low”.

#### Multilevel Cox regression analysis

The multilevel Cox proportional hazards regression model was employed with two levels, the first corresponding to the adolescents, and the schools at the second level [30]. The multilevel model allows to consider the correlation between individuals in the same cluster [31]; in this case, schools. The outcome was defined as the time from the beginning of the follow-up (January 2015) until the date on which the first symptoms of one of the arboviral diseases occurred. Non-case participants were censored at the end of the study (March 31, 2019) or when they became adults (age above 19 years, 11 months and 29 days).

The period of study was divided into 13 temporal blocks: three in 2015–2018 (January-April, May-August, September-December); and one in 2019 (January-March). In each of the temporal blocks, the BII values of the immediately previous period were associated. Thus, period 1, from January to April 2015, was classified according to the last measure of BII in 2014. Period 2, from May to August 2015, was associated with the first assessment of BII in 2015, and so on, until the final period of study.

The Intraclass Correlation Coefficient (ICC), also known as Variance Partition Coefficient (VPC), was obtained for a null model (without explanatory variables) and, subsequently, for the other models. The ICC or VPC quantifies the intra-cluster homogeneity degree (schools), indicating the proportion of outcome variance that can be attributed to the schools. An ICC that is equal to zero indicates that the observations do not depend on which cluster they are in, and an ICC that is equal to 1 indicates that all the variability of the outcome can be attributed to the clusters [31, 32]. The ICC was calculated using the equation below:

$$ICC=\frac{Va}{{Va+(\pi }^{2}/3)},$$

where *Va* is the variance at the school level, and *π*^2^/3 = 3.29 represents fixed variance at the individual level.

The Proportional Change in Variance (PCV) was calculated to verify how much of the variance found in the null model (V1) is explained by the variables included in a specified model (V2). The following formula was used:

$$PCV=\frac{V1-V2}{V1},$$

where *V*1 is the null model and *V*2 the model under analysis.

Bivariate analyses were performed to verify associations between the occurrence of arboviral diseases and the independent variables.

Six models were evaluated: (1) null model (without explanatory variables); (2) model with only individual variables; (3) model with individual and school variables (school attendance period and type of school); (4) model with school variables together with the school surroundings variables; (5) model with all variables; and (6) final model, including only the variables associated with the outcome in the bivariate analysis considering p-value <0.20. The premise of proportional hazards was tested to verify the presence of variation in the coefficient over time, using the Schoenfeld residuals test [33, 34]. We detected evidence of statistically significant violation of the proportional hazard assumption for municipality and it was removed from the final model (S2 Fig).

The *survival* and *coxme* packages of the R software were used. Associations were expressed in terms of Hazard Ratios (HR) and their respective 95%CI and p-values.

## Results

**Fig 1:**
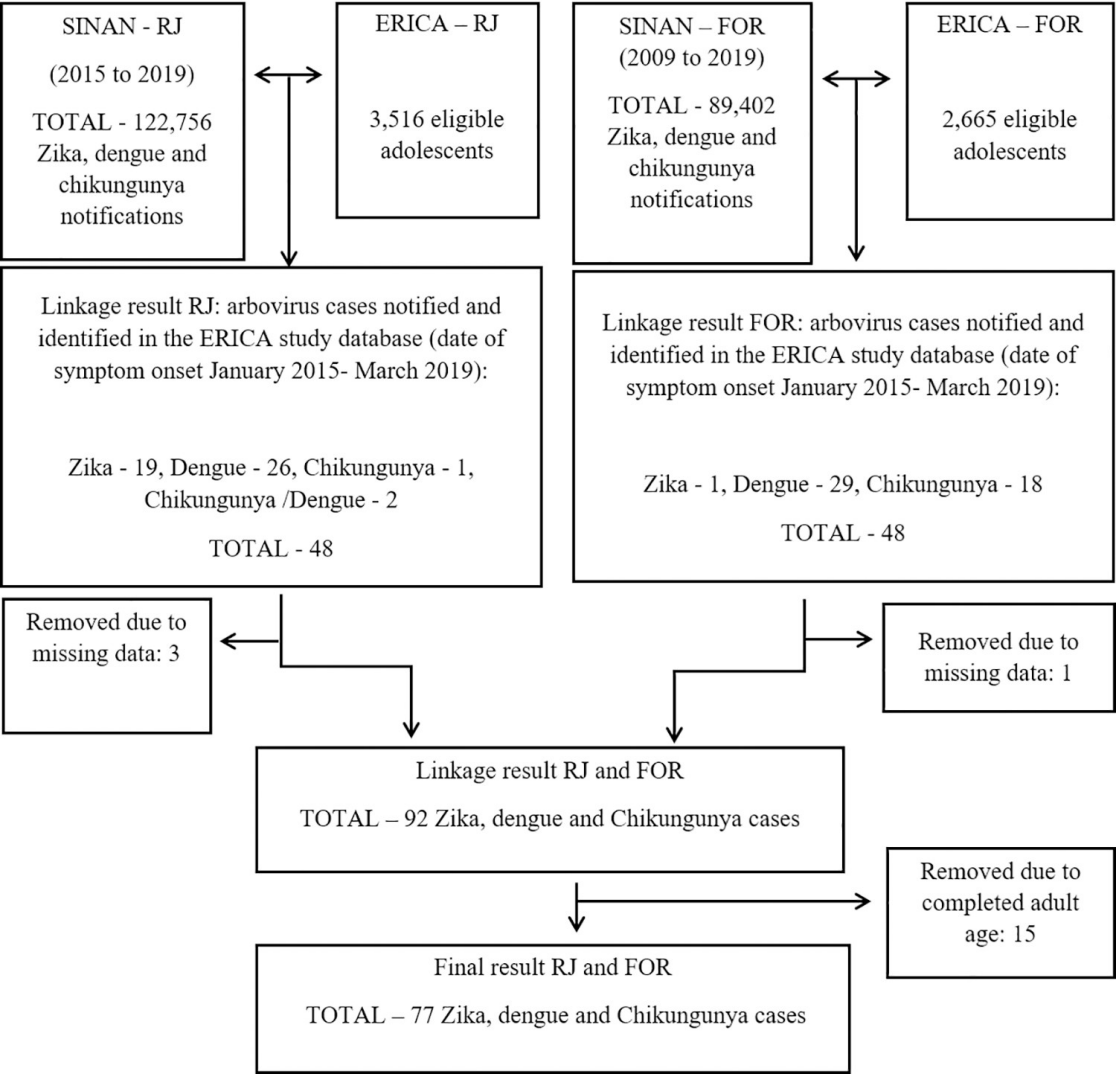
Flowchart of the data linkage for the cities of Rio de Janeiro/RJ and Fortaleza/CE.

The probabilistic data linkage for the city of Rio de Janeiro identified 50 notified cases of ZIKV, DENV, and CHIKV among 3,399 adolescents recruited and evaluated in 2013 in the ERICA study. Two of them were notified both for DENV and CHIKV with the same date of onset of symptoms and were computed only once for analysis purposes. Thus, we identified 48 cases of arboviral diseases from the city of Rio de Janeiro: 1 case of CHIKV, 19 of ZIKV, 26 of DENV, and 2 cases of DENV/CHIKV. In the manual data linkage for the city of Fortaleza, among 2,587 adolescents recruited and evaluated in 2013 in the ERICA study, 48 cases of arboviral diseases were found with onset of symptoms from January 2015 to March 2019: 29 cases of DENV, 1 of ZIKV, and 18 of CHIKV. Three adolescents from Rio de Janeiro and one from Fortaleza with more than one missing in sociodemographic characteristics were excluded. Fifteen participants, 13 from Rio de Janeiro and 2 from Fortaleza, became an adult before the date of the onset of symptoms and were censored. Thus, 77 cases of arboviral diseases from both cities were included in the analyses (Fig 1).

All adolescents in the ERICA cohort were 12 to 17 years old when they were evaluated in 2013. Of those with notification for ZIKV, DENV, or CHIKV, the majority attended school in the morning (95%) with an incidence (I) of 1.61 (I = 1.61%; 95%CI: 1.26–2.03), 36% with self-reported white skin color. The socioeconomic level of the majority of the cases was classified as very low (I = 2.09%; 95%CI: 1.35–3.08) (Table 1).

**Table 1:** Incidence and descriptive characteristics of the adolescents of the ERICA cohort.

	Total of Adolescents n = 5,986	Cases N = 77	Incidence % (95% CI)
**Gender**			
** Female**	3,322	43	1.29 (0.94–1.74)
** Male**	2,664	34	1.28 (0.88–1.78)
**Self-reported skin color**			
** White**	2,070	28	1.35 (0.90–1.96)
** Others**	3,916	49	1.25 (0.93–1.65)
**Age**			
** 12**	468	3	0.64 (0.13–1.87)
** 13**	934	11	1.18 (0.59–2.11)
** 14**	1,042	19	1.82 (1.10–2.85)
** 15**	1,331	22	1.65 (1.04–2.50)
** 16**	1,229	14	1.14 (0.62–1.91)
** 17**	982	8	0.81 (0.35–1.61)
**Socioeconomic level**			
** Very low**	1,198	25	2.09 (1.35–3.08)
** Low**	1,197	12	1.00 (0.52–1.75)
** Medium**	1,196	14	1.17 (0.64–1.96)
** High**	1,197	17	1.42 (0.83–2.27)
** Very high**	1,198	9	0.75 (0.34–1.43)
**School attendance period**			
** Morning**	4,524	73	1.61 (1.26–2.03)
** Afternoon**	1,462	4	0.27 (0.07–0.70)

A total of 100 schools (most of them public) participated in the study, 44 in Rio de Janeiro and 56 in Fortaleza. Most of the households in the census tracts to which the schools belonged presented water supply, garbage collection, and paved streets. Furthermore, the low presence of sewage network and high open-air sewage represented higher incidence (I) (I = 1.66%; 95% CI:1.15–2.32 and I = 2.12%; 95%CI: 1.33–3.21, respectively) (Table 2).

**Table 2:** Incidence and descriptive characteristics of the participant schools and of their surroundings—ERICA cohort.

	Schools n = 100	Adolescentes n = 5,986	Cases N = 77	Incidence % (95% CI)
Type of school				
** Private**	26	1,475	13	0.88 (0.47–1.51)
** Public**	74	4,511	64	1.42 (1.09–1.81)
**City**				
** Fortaleza**	44	2,587	43	1.66 (1.20–2.24)
** Rio de Janeiro**	56	3,399	34	1.00 (0.69–1.40)
**Presence of water supplied by the distribution network**				
** High**	88	5,329	66	1.24 (0.96–1.58)
** Low**	12	657	11	1.67 (0.83–3.00)
**Presence of sewerage network**				
** High**	65	3,934	43	1.09 (0.79–1.47)
** Low**	35	2,502	34	1.66 (1.15–2.32)
**Presence of collected garbage**				
** High**	90	5,430	70	1.29 (1.00–1.63)
** Low**	10	556	7	1.26 (0.51–2.59)
**Presence of sidewalk in the surrounding area**				
** High**	71	4,383	56	1.28 (0.97–1.66)
** Low**	29	1,603	21	1.31 (0.81–2.00)
**Presence of storm drain in the surrounding area**				
** High**	35	2,306	24	1.04 (0.67–1.55)
** Low**	65	3,680	53	1.44 (1.08–1.88)
**Presence of vegetation in the surrounding area**				
** High**	78	4,794	61	1.27 (0.97–1.63)
** Low**	22	1,192	16	1.34 (0.77–2.18)
**Presence of open-air sewage in the surrounding area**				
** High**	18	1,037	22	2.12 (1.33–3.21)
** Low**	82	4,949	55	1.11 (0.84–1.45)
**Presence of garbage accumulated in the surrounding area**				
** High**	11	704	5	0.71 (0.23–1.66)
** Low**	89	5,282	72	1.36 (1.07–1.72)
	**Schools- period n = 1,274**	**Adolescents- period n = 60,654**	**Cases n = 77**	**Incidence per 100 adolescents-period (95%CI)**
**Building Infestation Index (BII)**				
** Satisfactory**	757	36,122	37	0.10 (0.07–0.14)
** Alert**	495	23,592	35	0.15 (0.10–0.21)
** Risk**	22	940	5	0.53 (0.17–1.24)

The bivariate Cox regression analyses resulted in socioeconomic level, city, school attendance period, type of school, presence of sewerage network, open-air sewage and garbage accumulated in the surroundings, and BII as variables associated with the occurrence of arboviral diseases (p<0.2) and, therefore, eligible for inclusion in multivariate models (S2 Table).

The null Cox regression model (model 1, without variables) indicated that 5.6% (VPC = 5.60%) of the variability in the risk of arboviral diseases can be attributed to the schools. The model with only individual variables (model 2) identified that better socioeconomic housing conditions were associated with a lower risk of arboviral diseases. The model with individual variables and school variables (school attendance period and type of school; model 3) identified that, in addition to socioeconomic level, attending school in the afternoon was associated with a reduction in the risk of arboviral diseases. The model with school variables and school surroundings variables (model 4) detected that the risk of arboviral diseases increased in the schools located in areas with BII classified as “alert” and “risk” (Table 3).

**Table 3:** Measures of association (hazard ratios) obtained by multivariate Cox proportional hazards regression between arboviral diseases, socioeconomic/demographic characteristics and school characteristics of adolescents followed up by ERICA in the cities of Rio de Janeiro and Fortaleza, Brazil, from January 2015 to March 2019.

	Model 1 (null)	Model 2 (individual variables)		Model 3 (individual and school variables)		Model 4 (school and school surroundings variables)		Model 5 (all the variables)		Model 6 (final, selected variables—p<0.2)	
**Fixed Effects**		**HR (95%CI)**	**p-value**	**HR (95%CI)**	**p-value**	**HR (95%CI)**	**p-value**	**HR (95%CI)**	**p-value**	**HR (95%CI)**	**p-value**
**Age**		1.05 (0.9,1.24)	0.521	1.06 (0.91,1.25)	0.441			1.00 (0.85,1.18)	0.972		
**Sex**											
Female [ref.]		1.00	-	1.00	-			1.00	-		
Male		1.05 (0.67,1.65)	0.838	1.06 (0.68,1.67)	0.798			1.02 (0.65,1.6)	0.936		
**Color**											
White [ref.]		1.00	-	1.00	-			1.00	-		
Others		0.85 (0.53,1.36)	0.488	0.83 (0.52,1.34)	0.449			0.78 (0.49,1.25)	0.304		
**Socioeconomic level**											
Very Low [ref.]		1.00	-	1.00	-			1.00	-	1.00	-
Low		0.47 (0.24,0.94)	0.033	0.46 (0.23,0.91)	0.027			0.44 (0.22,0.89)	0.021	0.46 (0.23,0.91)	0.027
Medium		0.54 (0.28,1.05)	0.069	0.55 (0.28,1.06)	0.076			0.57 (0.29,1.1)	0.094	0.57 (0.3,1.11)	0.100
High		0.65 (0.35,1.2)	0.17	0.67 (0.35,1.26)	0.212			0.69 (0.37,1.31)	0.262	0.7 (0.37,1.32)	0.272
Very High		0.34 (0.16,0.75)	0.007	0.38 (0.17,0.87)	0.022			0.42 (0.18,0.97)	0.043	0.43 (0.19,0.99)	0.047
**Type of School**											
Private [ref.]				1.00	-	1.00	-	1.00	-	1.00	-
Public				1.29 (0.66,2.51)	0.451	1.48 (0.77,2.83)	0.236	1.31 (0.64,2.66)	0.457	1.08 (0.56,2.11)	0.811
**School Attendance Period**											
Morning [ref.]				1.00	-	1.00	-	1.00	-	1.00	-
Afternoon				0.17 (0.06,0.47)	< 0.001	0.16 (0.06,0.45)	< 0.001	0.15 (0.05,0.43)	< 0.001	0.17 (0.06,0.47)	< 0.001
**City**											
Fortaleza [ref.]						1.00	-				
Rio de Janeiro						0.84 (0.34,2.08)	0.704	0.86 (0.34,2.14)	0.741		
**Households with water distribution network**											
High [ref.]						1.00	-	1.00	-		
Low						1.58 (0.74,3.37)	0.239	1.66 (0.78,3.54)	0.191		
**Households with sewerage network**											
High [ref.]						1.00	-	1.00	-	1.00	-
Low						1.15 (0.64,2.05)	0.641	1.14 (0.64,2.05)	0.660	1.24 (0.72,2.12)	0.439
**Households with collected garbage**											
High [ref.]						1.00	-	1.00	-		
Low						0.86 (0.36,2.09)	0.747	0.83 (0.34,2)	0.672		
**Households with sidewalks in the surrounding area**											
High [ref.]						1.00	-	1.00	-		
Low						0.87 (0.42,1.79)	0.707	0.84 (0.4,1.74)	0.632		
**Households with storm drain in the surrounding area**											
High [ref.]						1.00	-	1.00	-		
Low						1.18 (0.45,3.1)	0.734	1.23 (0.47,3.24)	0.671		
**Households with vegetation in the surrounding area**											
High [ref.]						1.00	-	1.00	-		
Low						1.25 (0.56,2.79)	0.583	1.27 (0.57,2.85)	0.560		
**Households with open-air sewage in the surrounding area**											
High [ref.]						1.00	-	1.00	-	1.00	-
Low						0.69 (0.33,1.44)	0.321	0.69 (0.32,1.45)	0.323	0.67 (0.36,1.24)	0.205
**Households with accumulated garbage in the surrounding area**											
High [ref.]						1.00	-	1.00	-	1.00	-
Low						2.43 (0.94,6.31)	0.067	2.36 (0.9,6.18)	0.082	2.05 (0.8,5.29)	0.136
**Building Infestation Index (BII)**											
Satisfactory [ref.]						1.00	-	1.00	-	1.00	-
Alert						1.57 (0.94,2.61)	0.085	1.58 (0.94,2.65)	0.081	1.62 (0.98,2.7)	0.062
Risk						3.61 (1.19,10.95)	0.024	3.66 (1.2,11.14)	0.022	3.72 (1.27,10.9)	0.017
**Random Effects**											
Variance	0.1952	0.1619		0.0311		0.00008		0.00008		0.0004	
Std Dev	0.4418	0.4023		0.1763		0.0091		0.0091		0.0200	
ICC = VPC	5.6011	4.6903		0.9356		0.0025		0.0025		0.0121	
Variance explained PCV	ref.	17.06%		84.08%		99.96%		99.96%		99.80%	

In the final model (model 6), the highest socioeconomic level (“very high”), in comparison with the lowest level (“very low”), was associated with a 57% reduction (HR = 0.43; 95%CI: 0.19–0.99; p = 0.047) in the risk of arboviral diseases. The “low” socioeconomic level corresponded to a 54% reduction (HR = 0.46; 95%CI: 0.23–0.91; p = 0.027) when compared to the “very low” level. Attending school in the afternoon represented protection of 83% (HR = 0.17; 95%CI: 0.06–0.47; p<0.001) for incidence of arboviral diseases when compared to attending school in the morning. The risk of arboviral diseases was 1.6 and 3.7 times higher among students from schools located in census tracts with BII classified as “alert” (HR = 1.62; 95%CI: 0.98–2.70; p = 0.062) or “risk” (HR = 3.72; 95%CI: 1.27–10.9; p = 0.017), when compared to schools with a “satisfactory” BII (Table 3).

Comparing the null model (model 1) with the individual variables model (model 2) and with the individual and school variables model (model 3), we identified a gradual increase in the percentage of variance explained: from 17.06% in model 2 to 84,08% when the school variables were included. The inclusion of school surroundings variables (models 4, 5, and 6) explained more than 99% of the variance percentage attributed to the schools, resulting in a VPC<0.01%.

## Discussion

This study highlights the importance of sociodemographic risk factors for acquiring ZIKV, DENV, and CHIKV in a cohort of adolescents (ERICA) from the cities of Rio de Janeiro/RJ and Fortaleza/CE, respectively, in the Southeast and Northeast regions of Brazil, recruited in 2013–2014 and followed up in the period from January 2015 to March 2019 using notification records. Among the significant results, high building infestation indexes (BII) by *Ae*. *aegypti* in the area surrounding the schools and lower socioeconomic conditions were associated with an increased risk of arboviral diseases.

Larval sampling, expressed by the BII has been used to identify areas at higher risk of arboviruses transmission and thus timely targeting of *Ae*. *aegypti* control activities [27, 35]. An association between increased risk of DENV and indicators of infestation by the vector *Ae*. *aegypti* has been shown by many studies [14, 15], but other studies have not found such an association [14, 36, 37], probably reflecting the diversity of methods employed for entomological assessment and the evaluation of transmission contexts.

The present study found that adolescents living in precarious socioeconomic situations present a higher risk of acquiring ZIKV, DENV, or CHIKV, and greater protection was observed with the gradual improvement in the socioeconomic level. Lower socioeconomic indicators have been related to high incidence rates of DENV [36], ZIKV [10], and CHIKV [17]. In contrast, some studies have verified an association between better socioeconomic conditions with a higher risk of ZIKV[17]. Each one of the cited studies captured the socioeconomic situations differently. The socioeconomic level indicator used in our study was generated through a PCA based on household characteristics and asset ownership and sought to capture, in a coherent way, the expected relations between those phenomena [29].

The utilization of the school as a possible proxy for place of transmission has been little explored and is an innovative aspect of our study. A relevant result related to this aspect was the protective effect found when the adolescent attended school in the afternoon (HR = 0.17; 95%CI: 0.06–0.47). Adolescents spend a large part of the day outside their homes, in the school environment, which coincides with the times when the vectors of arboviral diseases prefer to feed on blood, with peaks at the beginning of the morning and the end of the afternoon. A study in Colombia identified a high frequency and infection rate by *Ae*. *aegypti* in schools, showing that they need to be included in the activities of health education, surveillance, and control of these infections [21]. Another study conducted in Northeast Region of Brazil was able to detect DENV-3 in pools of *Aedes* mosquitoes collected from several non-residential premises, including public schools, where the authors also observed the highest abundance of *Aedes* mosquitoes [22]. The authors concluded that places of high human influx, such as health care facilities and schools, should always be considered for the continuous monitoring of *Aedes* mosquitoes’ infestation in order to guide local control actions.

Factors related to the quality of households and the socioenvironmental characteristics of the regions surrounding them are considered determinants for the occurrence of diseases transmitted by vectors. The main factors are high population density, inadequate habitations, precarious water supply, and waste disposal, high urban mobility, and climate variables like temperature and rainfall, all of them correlated with each other and with a greater infestation of *Ae*. *aegypti* [12, 27]. The presence of storm drains has been related to the abundance of *Ae*. *aegypti* and *Aedes albopictus* [38], and living near storm drains, to a higher incidence of DENV [39]. A study in Colombia, for example, has shown that the introduction of chemical products for vector control in storm drains considerably reduced the number of vector larvae and the number of DENVcases in the region [40]. The presence of vegetation has also been indicated as a factor that can increase the odds of arboviral diseases [16], although other studies have suggested there is no relationship between vegetation cover and the risk of ZIKV and CHIKV [17]. The vectors *Ae*. *aegypti* and *Ae*. *albopictus* can coexist in peri-urban regions, with *Ae*. *aegypti* having a stronger association with urbanization and *Ae*. *albopictus* with wooded areas [41, 42]. In our study, the school surroundings variables referring to basic sanitation, garbage collection, vegetation cover, paved streets, and the presence of storm drains were not associated with the incidence of arboviral diseases. One possible explanation for the occurrence of these results is the study’s low power to detect significant associations. It is also possible that the most vulnerable areas, perhaps because of their history of DENVoccurrence, have been prioritized in vector control, reducing the risk in these locations. In these contexts, the school environment may not be an adequate proxy for the place of infection.

This study has some limitations. As the diagnosis of DENV, ZIKV, and CHIKV can be a challenge due to similarity in symptoms [43], it is possible that some cases were mistakenly classified, mainly at the beginning of the ZIKVepidemic in 2015 [44–46]. Nevertheless, since the three viruses are transmitted by the same vector, integrated intervention approaches to the three infections are desirable and justify a joint analysis. Also, asymptomatic infections and mild cases are underreported in SINAN. Yet, this is a common limitation in arbovirus analysis, particularly regarding asymptomatic cases. Lastly, it was impossible to assess the context of students’ dwelling place due to the lack of residential addresses. However, it is possible to assume that, at least in part, the environmental characteristics of the area surrounding the schools are similar to those of the households. This occurs because data from different Brazilian cities indicate that more than half of the households are located in areas relatively close to schools [47–50]. In fact, the school variable “school attendance period”, together with the school surroundings variable (BII), explained the largest part of the variance attributed to the schools. Other potential limitations were the non-incorporation in the model of the periods in which the students were on school breaks and holidays; and the need for more information on different behaviors of adolescents, such as those younger spending more time at school than older age groups. Such data would be necessary for helping identify specific groups and periods of higher risk. However, individual demographic variables were not associated with the risk of disease, suggesting that, in this particular context, socioeconomic and environmental factors related to the home and the school are likely to be more relevant than individual and behavioral characteristics.

Many methods have been employed to control arboviral diseases. However, in the absence of an efficacious vaccine [51], vector control remains the most used approach. New techniques that modify the survival and reproduction capacity of the mosquito are being developed, such as the use of *Wolbachia*-infected *Aedes* spp. [52]. However, the scalability and sustainability of those novel approaches is yet to be proven, and thus vector control with insecticides and, health education strategies have been the most commonly used strategies in Brazil [27].

The results of this study indicate that a substantial portion of the variation in the risk of arboviral diseases among adolescents is associated with the school environment. In this context, adolescents’ lower socioeconomic conditions, in association with high building infestation indexes *Ae. aegypti* in the region where the schools are located, and attending school in the morning period, were the main risk factors for ZIKV, DENV and CHIKV. Our findings show that, in addition to the household environment, other contexts, such as schools, need to be improved in the planning of control actions.

## Supporting information

S1 Fig Percentages of the variances explained by each dimension represented by the Scree Plot as a result of the PCA for socioeconomic variables, cities of Rio de Janeiro/RJ and Fortaleza/CE. (TIF)

S2 Fig Schoenfeld residuals Test performed to test the premise of proportional hazards for the variables selected in the final model. (TIF)

S1 Table Loadings of the variables in the first components of the principal components analyses used to generate the socioeconomic variable, Rio de Janeiro/RJ and Fortaleza/CE. (XLSX)

S2 Table Measures of association (hazard ratios) obtained by the bivariate Cox proportional hazards regression between arboviral diseases, socioeconomic/demographic and school characteristics of adolescents followed up by ERICA in the cities of Rio de Janeiro and Fortaleza, Brazil, January 2015 to March 2019. (XLSX)
