# Supplementary figures and images for: Sociodemographic and environmental factors associated with dengue, Zika, and chikungunya among adolescents from two Brazilian capitals
Source: PLoS Negl Trop Dis. 2023 Mar 16;17(3):e0011197. doi: 10.1371/journal.pntd.0011197 (PMC10047540; doi:10.1371/journal.pntd.0011197)

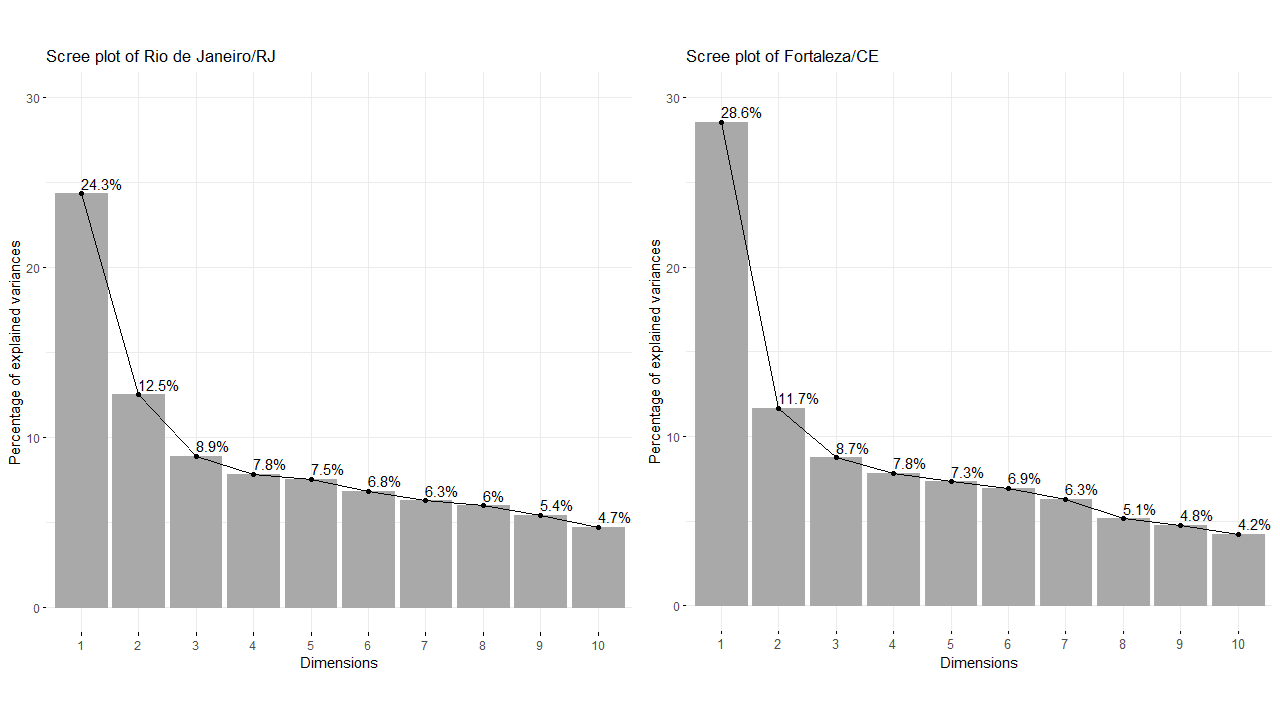

Supplement: S1 Fig — (TIF) [file pntd.0011197.s001.tif]

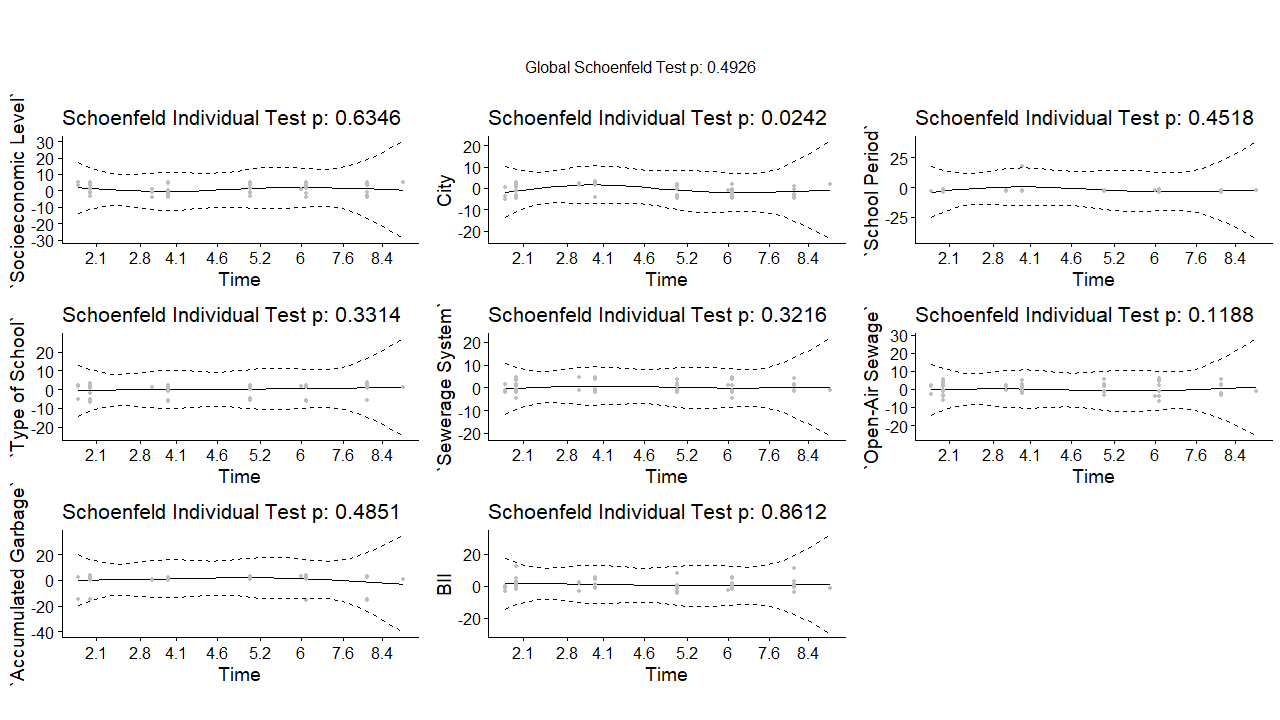

Supplement: S2 Fig — (TIF) [file pntd.0011197.s002.tif]
